# ^19^F-NMR reveals substrate specificity of CYP121A1 in *Mycobacterium tuberculosis*

**DOI:** 10.1016/j.jbc.2021.101287

**Published:** 2021-10-08

**Authors:** Christopher S. Campomizzi, George E. Ghanatios, D. Fernando Estrada

**Affiliations:** Department of Biochemistry, Jacobs School of Medicine and Biomedical Science, University at Buffalo, Buffalo, New York, USA

**Keywords:** Cytochrome P450, NMR, CYP121A1, ^19^F, fluorine, BTFA, 3-bromo-1,1,1-trifluoroacetone, CYPs, cytochrome P450, cYY, dicyclotyrosine, DMSO, dimethyl sulfoxide, Mtb, *Mycobacterium tuberculosis*, TB, tuberculosis

## Abstract

Cytochromes P450 are versatile enzymes that function in endobiotic and xenobiotic metabolism and undergo meaningful structural changes that relate to their function. However, the way in which conformational changes inform the specific recognition of the substrate is often unknown. Here, we demonstrate the utility of fluorine (^19^F)-NMR spectroscopy to monitor structural changes in CYP121A1, an essential enzyme from *Mycobacterium tuberculosis*. CYP121A1 forms functional dimers that catalyze the phenol-coupling reaction of the dipeptide dicyclotyrosine. The thiol-reactive compound 3-bromo-1,1,1-trifluoroacetone was used to label an S171C mutation of the enzyme FG loop, which is located adjacent to the homodimer interface. Substrate titrations and inhibitor-bound ^19^F-NMR spectra indicate that ligand binding reduces conformational heterogeneity at the FG loop in both the dimer and in an engineered monomer of CYP121A1. However, only the dimer was found to promote a substrate-bound conformation that was preexisting in the substrate-free spectra, thus confirming a role for the dimer interface in dicyclotyrosine recognition. Moreover, ^19^F-NMR spectra in the presence of substrate analogs indicate the hydrogen-bonding feature of the dipeptide aromatic side chain as a dicyclotyrosine specificity criterion. This study demonstrates the utility of ^19^F-NMR as applied to a multimeric cytochrome P450, while also revealing mechanistic insights for an essential *M. tuberculosis* enzyme.

Cytochromes P450 (CYPs) are a superfamily of heme-coordinated enzymes that are characterized in part by a highly conserved protein fold despite sharing as little as 10% sequence identity (1). CYPs are ubiquitous in nature and mediate catalysis for a wide range of substrates, which include xenobiotic and endogenous reactions in mammals and complex biosynthetic reactions in plants and bacteria (2). An accepted feature of CYP function is the requirement for subtle yet meaningful changes in the enzyme structure, whether to accommodate chemically diverse substrates (3, 4), to interact with a redox partner, or to facilitate a combination of both of these events (5, 6, 7).

NMR spectroscopy is a valuable tool that in theory is well suited to detect conformational equilibria in CYP structures. However, multidimensional NMR techniques are difficult to apply broadly to CYPs because of their large relative size (larger than 45 kDa) and their tendency to form multimeric complexes in solution. In this work, we demonstrate an application of fluorine (^19^F)-NMR using site-specific labeling of functional CYP121A1 dimers (90 kDa) from *Mycobacterium tuberculosis* (Mtb). The ^19^F nucleus exhibits similar sensitivity for NMR detection to that of proton nuclei. Moreover, because ^19^F is not naturally incorporated into proteins, ^19^F labeling results in one-dimensional spectra that are greatly simplified by a reduction in spectral crowding (8, 9).

Mtb is the pathogenic bacterium that causes tuberculosis (TB). According to the World Health Organization, before the COVID-19 pandemic, TB represented the single highest cause of death by infection worldwide. A more dire concern is the rise of multidrug-resistant forms of the disease, which account for approximately one-half million infections per year and underscore the need to develop novel anti-TB drugs (10). Mtb contains a disproportionately high number of CYP genes, with approximately 200 times the CYP gene density found in humans (11). Of these, CYP121A1 has been reported as essential because attempts to delete the corresponding gene (*rv2275*) result in nonviable bacteria (12). CYP121A1 mediates a rare phenol-coupling reaction of the dipeptide dicyclotyrosine (cYY) to produce mycocyclosin, a product of undetermined function (13). Despite this, inhibition of CYP121A1 remains a potential strategy in the treatment of TB and multidrug-resistant forms of the disease (14, 15, 16).

CYP121A1 is known to form stable dimers in solution (17). However, until recently, the significance of dimer formation was unclear. In a recent study, we mapped the nonpolar solution dimer interface of CYP121A1 to the F and G α-helices, which correlate to the distal or substrate-binding surface of CYPs (Fig. 1*A*) (18). Protein docking and cross-linking indicated that CYP121A1 molecules are arranged as dimers along the distal surface and rotated by approximately 180°. Notably, the solution dimer interface had not been detected in the crystallographic data. Subsequently, mutagenesis of residues Ile-166 and Ile-180 to alanine was found to reduce the hydrophobic character of the distal surface, resulting in an equilibrium that strongly favors the monomeric form of the enzyme, as opposed to the dimer-predominant equilibrium of native CYP121A1. The double mutant was then found to have significantly reduced catalytic activity, likely as a result of compromised substrate specificity (18). Here, we use ^19^F-NMR to monitor ligand binding in CYP121A1 from the perspective of the intervening FG loop (Fig. 1*A*) (18). Remodeling and dynamics of the FG loop are centrally important for gating substrate access to the buried active site in mammalian and bacterial CYPs (3, 4, 19, 20, 21, 22, 23, 24, 25). Despite the expected changes in this region, crystal structures of the enzyme in complex with the substrate, the inhibitor fluconazole, or in a ligand-free form reflect the same general positioning of the FG loop (Fig. 1*A*). In contrast, our prior (albeit ligand-free) ^19^F-NMR spectra of the FG loop indicated the presence of at least three distinct conformations (18).

**Figure 1:**
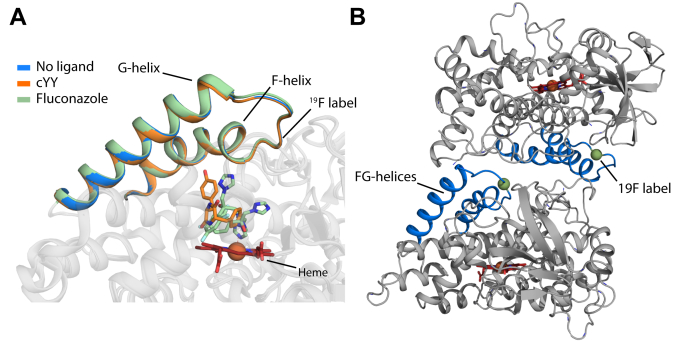
**Overview of CYP121 structures highlighting the FG region.***A*, modeled CYP121A1 dimer interface with the FG helices (*blue*) and the CF_3_-labeled site (*green*) highlighted at the interface. *B*, substrate-free (*blue*), cYY-bound (*orange*), and fluconazole-bound (*green*) crystal structures (PDB IDs: 3G5F, 3G5H, and 2IJ7, respectively (13, 35)). ^19^F, fluorine.

Here, we report that titration of cYY into ^19^F-labeled CYP121A1 reduces conformational heterogeneity at the FG loop. Comparative ^19^F-NMR spectra of the enzyme monomer and dimer also indicate that the dimer interface facilitates the formation of distinct ligand-bound and ligand-free conformations of the FG loop, which are notably absent in the monomer. We discuss these findings in the context of specific substrate recognition by a modeled homodimer of the enzyme. To our knowledge, this is the first application of ^19^F-NMR to interrogate changes in CYP enzyme structure and thereby serves to demonstrate the utility of this approach for the study of multimeric CYP complexes.

## Results

### The FG loop exhibits conformational heterogeneity

^19^F-NMR spectra presented in the previous study suggested that the FG loop of the CYP121A1 dimer is heterogeneous. The S171C mutant labeled with 3-bromo-1,1,1-trifluoroacetone (BTFA) displayed three distinct resonances (18). Having confirmed that neither the S171C mutation nor BTFA labeling significantly disrupts the function of the enzyme (Fig. S1), here, we set out to use ^19^F labeling of the FG loop as a way to monitor changes in its conformational heterogeneity. We designated the different resonances as *f*_*1*_ (free-1), *f*_*2*_, and *f*_*3*_. Peak *f*_*1*_ (−83.65 ppm) is a broad resonance that overlaps partially with peak *f*_*2*_ (−84.20 ppm), and peak *f*_*3*_ (−85.87 ppm) is a lower intensity signal located upfield of the major *f*_*2*_ peak.

**Figure 2:**
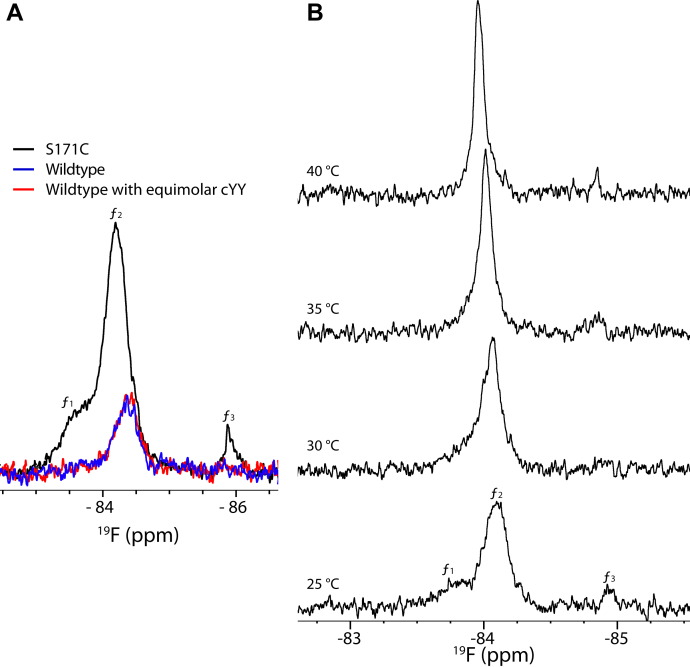
**Characterization of**^**19**^**F-NMR spectra of the CYP121A1 FG loop.***A*, overlaid spectra of the ^19^F-labeled WT protein and protein containing the S171C mutation. *B*, four spectra were acquired at 5 °C increments from 25 °C to 40 °C. In the 25 °C spectra, peak centers (peaks *f*_1−3_) are at −83.65, −84.20, and −85.87 ppm, respectively. ^19^F, fluorine.

First, to confirm that the observable signal corresponds to the FG loop, we acquired a spectrum of WT CYP121A1 without the additional cysteine (Fig. 2*A*). The spectrum contains a broad, low-intensity signal that appears to overlap with the more prominent signal from the S171C-labeled site. This is consistent with LC-MS/MS data (data not shown) that indicate that some labeling does occur at Cys-51 but with the majority of labeling occurring at S171C of the FG loop. However, we also found that addition of an equimolar amount of the cYY substrate does not affect this signal (Fig. 2*A*, red trace). These data together indicate that all of the spectral perturbations reported later in this study originate from the S171C site on the FG loop.

Next, to confirm that these signals represent distinct conformations of the FG loop, we acquired spectra of separate samples at incremental temperatures. Owing to the instability of the sample at higher temperatures, the spectra in Figure 2 represent data from shorter acquisitions than were used in later titrations. As expected, there is a gradual downfield shift in chemical shift values of all peaks. Notably, peak *f*_*1*_ broadens, then appears to merge with peak *f*_*2*_, which becomes narrower, likely in response to temperature-induced motions of the FG loop. These spectra indicate that resonances *f*_*1*_ and *f*_*2*_ belong to the same ^19^F-labeled population of CYP121A1, as indicated by increased exchange between states when the acquisition temperature is increased. Interestingly, peak *f*_*3*_ sharpens, but does not merge with peaks *f*_*1*_ and *f*_*2*_, suggesting that this resonance represents a distinct population of CYP121A1 that does not exchange with the primary population. Notably, a corresponding peak *f*_*3*_ is not present in the spectra of the monomeric protein, which may indicate that at the concentrations used, the dimeric enzyme forms a higher-order assembly. Follow-on data were acquired at 25 °C and at the lower concentration of 125 μM (Fig. S2). At this concentration, peak *f*_*3*_ is not detectible, and the distribution of peaks *f*_*1*_ and *f*_*2*_ is largely unchanged. In aggregate, these spectra indicate that at an ambient temperature and 250 μM, the predominant form of the enzyme remains as a homodimer and that the principal contributor to the ^19^F spectrum is the conformationally heterogeneous FG loop of the enzyme.

### Substrate binding reduces conformational heterogeneity of the FG loop

We recently examined the functional role of the CYP121A1 dimer by generating an I166A_I180A double mutation that favors a monomeric form of the enzyme (18). We observed that the greater dispersion of ^19^F-NMR resonances in the dimer (of approximately 2.5 ppm compared with 0.5 ppm in the monomer) indicates that the dimer interface contributes structural order to the intervening FG loop. Here, we titrated samples of ^19^F-labeled monomeric and dimeric CYP121A1 with the substrate to determine the effect that substrate binding in either form confers on the conformations of the FG loop.

**Figure 3:**
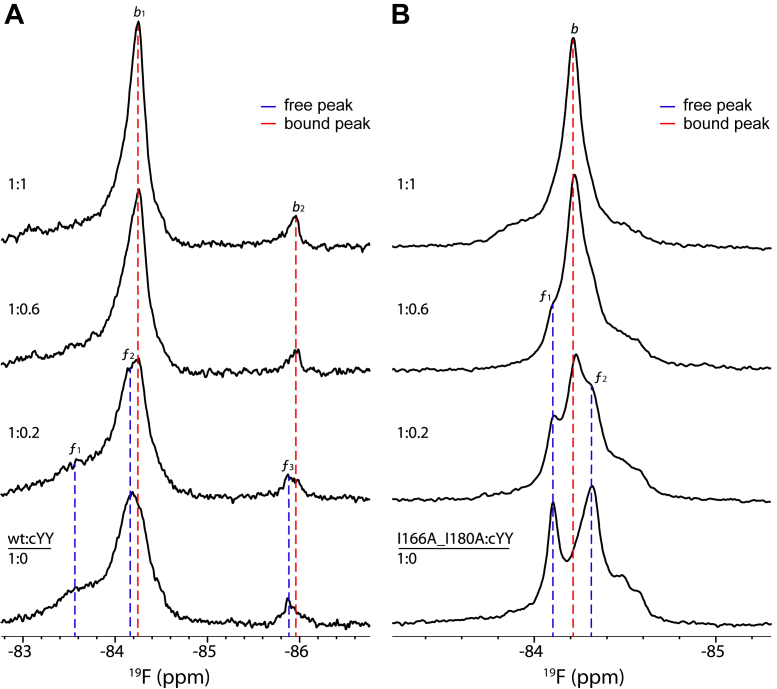
**cYY titrations into**^**19**^**F-labeled CYP121A1.** cYY titrations into the ^19^F-labeled WT/dimer (*A*) and I166A_I180A/monomer (*B*) as represented by the incremental ratios of CYP:cYY. All spectra are normalized for intensity. ^19^F, fluorine.

Upon titration with cYY, peak *f*_*1*_ decreases in intensity. This is accompanied by changing contours of the *f*_*2*_ peak in which its maximum changes from −84.20 ppm to −84.25 ppm, along with an increase in its relative intensity. The center of the smaller peak *f*_*3*_ also redistributes from −85.87 ppm to −85.97 ppm. By the equal-molar (1:1) protein to substrate ratio, the spectrum reflects two primary bound conformations (top trace in Fig. 3*A*). Notably, close examination of the cYY-bound spectra indicates that both of these peaks, designated as *b*_*1*_ (bound-1) and *b*_*2*_, are already represented as minor or broad resonances in the ligand-free spectrum and that substrate-induced enhancement of these signals occurs concomitantly with loss of the ligand-free peaks, consistent with an interaction occurring at a slow chemical exchange. Interestingly, further titrations of cYY to include 1:2 and 1:4 ratios (Fig. S3*A*) show minimal change to the substrate-bound spectrum, thereby suggesting that the saturation of the interaction occurs near an equal-molar ratio.

Next, we also looked at the effect of cYY titration on the CYP121A1 monomer (I166A_I180A) that also contains the S171C mutation for ^19^F labeling. In the ligand-free sample, we detected two primary narrow peaks at −84.10 ppm and −84.32 ppm (Fig. 3*B*). Although there appears to be more heterogeneity in the FG loop in the monomer, upon cYY addition, all peaks decrease in intensity and a single peak (*b*) emerges at −84.22 ppm. Similar to the dimeric protein, 1:1, 1:2, and 1:4 titration points show little further binding (Fig. S3*B*). However, we should note that although the substrate-bound conformation (*b*_*1*_) in dimeric CYP121A1 is already present as a shoulder to the main ligand-free resonance, this does not appear to be the case for the monomer. Therefore, the ordered structure provided by the dimer interface likely facilitates substrate recognition by populating a bound conformation in the ligand-free form. Next, we quantified changes in peak intensities for the *b1* and *b* peaks, respectively, to estimate dissociation constants for the ^19^F-NMR titration. These saturation plots are represented in Figure S3*C*. Here, we observed that a reduction in dimerization does not disrupt the affinity of CYP121A1 for cYY as measured from the FG loop, despite a net loss of 75% of the substrate binding when measured by difference absorbance spectra (18).

### Substrate binding measured by ^19^F-NMR and UV-Vis

**Figure 4:**
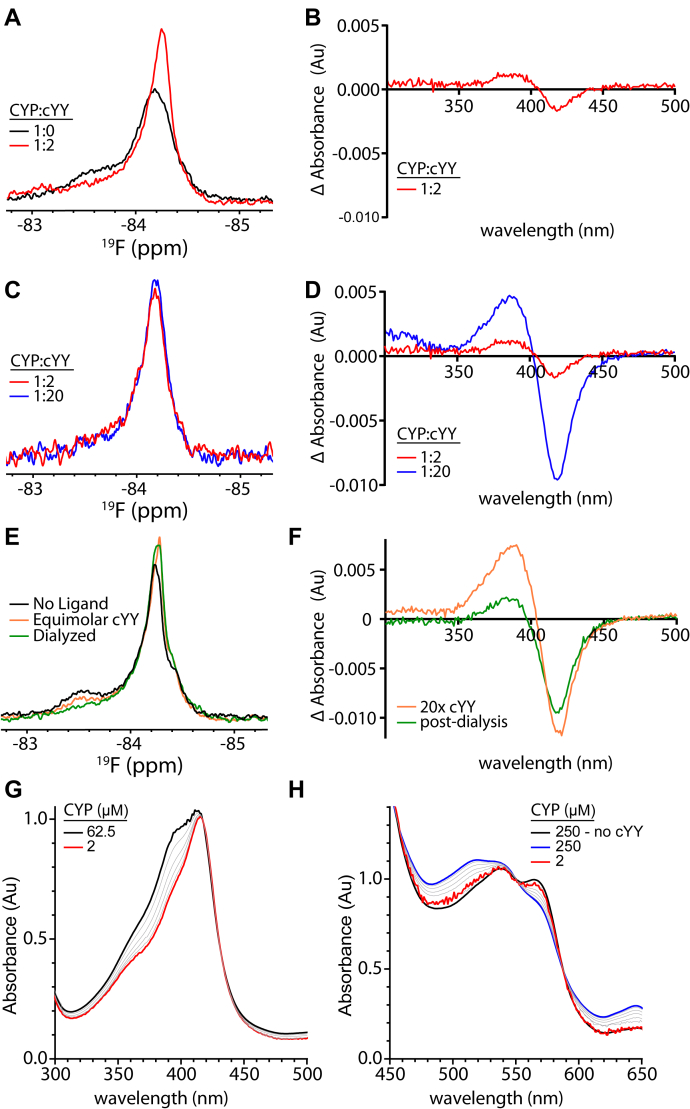
**Characterization of cYY binding in CYP121A1 measured by the FG loop.**^19^F-NMR spectra of 1:0 *versus* 1:2 ratios (*A*) and 1:2 *versus* 1:20 ratios (*C*) of CYP:cYY. UV-visible absorbance difference spectra of 1:2 ratio (*B*) and 1:2 *versus* 1:20 ratios (*D*) of CYP:cYY. *E*, ^19^F-NMR spectra of ligand-free (*black*), cYY-bound (*orange*), and dialyzed (*green*) CYP121A1. *F*, UV-visible absorbance difference spectra of high-spin shifted (*orange*) and subsequently dialyzed CYP121A1 (*green*). UV-absorption spectra of dilutions of CYP121A1 in the presence of equimolar cYY, monitoring changes in the Soret band (*G*) and the Q-band spectra (*H*).

A hallmark of substrate binding in CYPs is the displacement of a heme-coordinated water molecule in the active site, which results in a blue shift of the heme-iron absorption (Soret) peak from a low-spin toward high-spin state (26, 27, 28). The affinity of cYY using such spectral-binding assays is approximately 10 μM (18). However, upon titrating cYY by ^19^F-NMR, we observed that the enzyme-to-substrate ratios that induce significant perturbations of the one-dimensional spectrum also display only a minor change in the corresponding absorption spectra. For example, the NMR spectrum of the protein in a 1:2 ratio shows a considerable change in the FG loop (Fig. 4*A*). By comparison, the corresponding ratio reflects only a marginal amount of high-spin signal by absorbance (Fig. 4*B*).

To explore whether the heme spin-state change is reflected at all in the NMR spectra, we also acquired the corresponding data using a 20-fold concentration of cYY (Fig. 4, *C* and *D*). A 1:20 ratio creates measurable high-spin shift at the heme iron, indicating the presence of the substrate bound to a population of the protein as traditionally measured by absorption spectroscopy. However, we detected only a minor change in the ^19^F-NMR spectrum between the 1:2 and the 1:20 titration points. Next, we asked whether the effect on the NMR spectrum is reversible. A sample of labeled S171C dimer was combined with equimolar cYY, diluted to 4 μM, and the dissociated substrate was removed by a 3-h dialysis against a 50-fold volume of the NMR buffer. The NMR spectrum of the reconcentrated sample shows that a significant amount of the enzyme remains bound to cYY (Fig. 4*E*), consistent with the slow dissociation of the substrate. In contrast, when an analogous experiment was performed using 20× cYY and the absorption spectra analyzed, the retention of cYY was markedly less, with the majority of the high-spin form reverting to the low-spin form (Fig. 4*F*).

Thus, measurements of substrate binding by each method appeared to be in disagreement with the ^19^F-NMR data, suggesting the presence of a higher affinity interaction with the enzyme. However, we should note that NMR and absorbance data were collected using protein concentrations of 250 μM and 1 μM, respectively. To address whether the enzyme–substrate interaction is subject to a concentration dependence, we measured absorbance changes after a series of dilutions from a high-concentration sample of CYP121A1 that had been preincubated (1:1) with cYY. We observed that at higher concentrations, a detectible proportion of spectral change exists, as noted by Soret band measurements up until 60 μM and Q-band measurements up until 250 μM (Fig. 4*G*). These data suggest that at the high concentrations necessary for NMR acquisition, an equimolar amount of the substrate is sufficient to generate appreciable high-spin binding. Therefore, substrate-induced perturbations of the ^19^F-NMR spectra appear to be concurrent with changes in heme absorbance. Although this finding does not rule out the possibility of a high-affinity interaction (as suggested previously by NMR spectra of the dialyzed samples [Fig. 4*E*]), it does make comparisons of substrate affinity by NMR and absorbance less clear.

### Imidazole titration of the ^19^F-NMR spectrum

**Figure 5:**
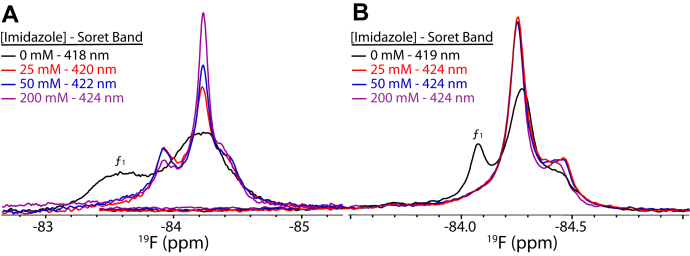
**Imidazole titrations into**^**19**^**F-labeled CYP121A1.**^19^F-NMR spectra of dimeric (*A*) and monomeric (*B*) CYP121A1 dialyzed against buffers containing 0 mM (*black*), 25 mM (*red*), 50 mM (*blue*), and 200 mM (*purple*) imidazole. ^19^F, fluorine.

Having established the pattern of perturbations on the ^19^F-NMR spectrum for a specific interaction with cYY, we then wanted to characterize the effect using imidazole as a nonspecific ligand. The aqueous solubility of imidazole combined with its interaction with the CYP heme make it a useful nonspecific ligand to saturate the interaction at higher protein concentrations required for NMR, while also providing a comparison between changes in the NMR spectra and changes in heme absorbance. In the dimer, we observed a stepwise shift in the ^19^F-NMR spectra that resulted in two remaining conformations of the FG loop (Fig. 5*A*). Of note is that changes in the NMR spectra correlated closely with a stepwise shift in the Soret band of the protein when titrated with the same molar ratio of imidazole.

Interestingly, in the I166A_I180A mutant, we observed rapid saturation for both of the NMR and wavelength absorption outputs, with significant changes in the ^19^F-NMR spectrum occurring at the lowest concentration measured (Fig. 5*B*). This is consistent with the higher affinity of the monomeric enzyme for imidazole, previously reported (18), in which the monomer binds imidazole with a 10-fold increase in affinity. We should also note that, in contrast to the titrations with the specific ligand (cYY) (Fig. 4), absorbance data recorded on diluted NMR samples do reflect changes in absorption in a way that more closely correlates with the amount of compound interacting at the heme. This indicates that the imidazole-bound samples are not subject to a concentration-dependent effect as was observed with cYY.

### Interactions with dipeptide substrate analogs

**Figure 6:**
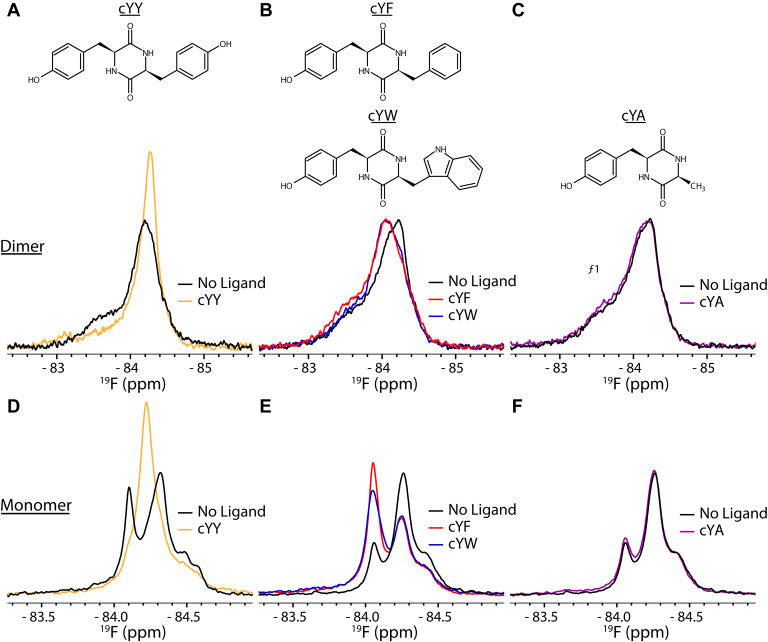
^**19**^**F-NMR spectra of CYP121A1 with cYY analogs.** cYY binding (*A*) is compared with the binding to analogs cYF and cYW (*B*) and cYA (*C*) for the ^19^F-labeled samples of dimeric (S171C) CYP121A1. Corresponding spectra are shown (*D–F*) for monomeric (S171C_I166A_I180A) CYP121A1. ^19^F, fluorine.

When titrated with imidazole, the NMR spectral changes are distinct compared with those observed with cYY. This may indicate that the unique perturbations with cYY are due to a specific recognition of CYP121A1 for the substrate. To more closely identify specificity criteria for cYY, we also acquired ^19^F-NMR spectra in the presence of a set of dipeptide analogs of the substrate (Fig. 6). The analogs (a gift from the group of Dr Pascal Belin) consist of single side-chain substitutions of cYY; substituting a tyrosine with alanine (cYA) to remove the second aromatic group, with phenylalanine (cYF) to remove the hydrogen bonding capability of the tyrosine hydroxyl group, and with tryptophan (cYW) to retain the hydrogen-bonding capability of the indole amide. These compounds have been shown to cocrystallize with the enzyme in a similar manner to cYY despite having a lower affinity by the spectral binding assay (29). Owing to the low aqueous solubility of cYF, all NMR spectra were acquired using no more than an equimolar ligand concentration to ^19^F-labeled CYP121A1.

As previously shown (Figs. 3 and 6*A*), an equimolar addition of cYY causes an overall increase in the peak *f*_*2*_ intensity, along with an upfield shift of the peak center and concurrent loss of the broad *f*_*1*_ peak. Interestingly, equimolar additions of cYF and cYW cause peak *f*_*2*_ to shift in the opposite direction (downfield), suggesting these compounds can interact with the enzyme, albeit in a manner different from that of cYY (Fig. 6*B*). An equimolar addition of cYA, on the other hand, shows no perturbations from the ligand-free spectrum (Fig. 6*C*). We also acquired corresponding data for the I166A_I180A mutation (Fig. 6, lower panel). Here, we observed that cYF and cYW also induce an opposite-field perturbation of the central resonance, with what appears to be an increase of a minor preexisting conformation. This outcome is again unique from that observed with the cYY interaction with the I166A_I180A mutant, where the substrate-bound peak is not already present. Meanwhile, the addition of cYA, as with the dimeric protein, does not perturb the spectrum.

Our interpretation of these data is that, although the presence of both aromatic groups appears to be a minimal requirement for ligand binding, the presence and positioning of the second tyrosine hydroxyl as a hydrogen bond donor represent a critical requirement for fully specific binding as measured by ^19^F-NMR of the FG loop. Moreover, the increased mobility of the FG loop when the dimer is disrupted (lower panel, center) likely results in a nonspecific conformation that is enhanced in the presence of the two aromatic cYY analogs.

### The CYP121A1 dimer interface confers differential binding near the FG loop

As a further characterization of general ligand binding near the FG loop, we also acquire ^19^F-NMR spectra in the presence of nonspecific CYP inhibitors of variable size and chemistry, using the dimeric and monomeric forms of the enzyme. ^19^F-labeled protein was dialyzed into the NMR buffer supplemented with 200 μM of each azole ligand. As measured by absorption spectroscopy, azole-containing compounds (type-II ligands) are expected to coordinate directly to the heme iron and are thus characterized by a red-shift change in the Soret band. By this measure, some compounds introduced by dialysis did not appear to fully bind to the enzyme dimer, with absorption peak changes going from 417 nm (before dialysis) to 418 nm and 421 nm when dialyzed with ketoconazole and econazole, respectively.

**Figure 7:**
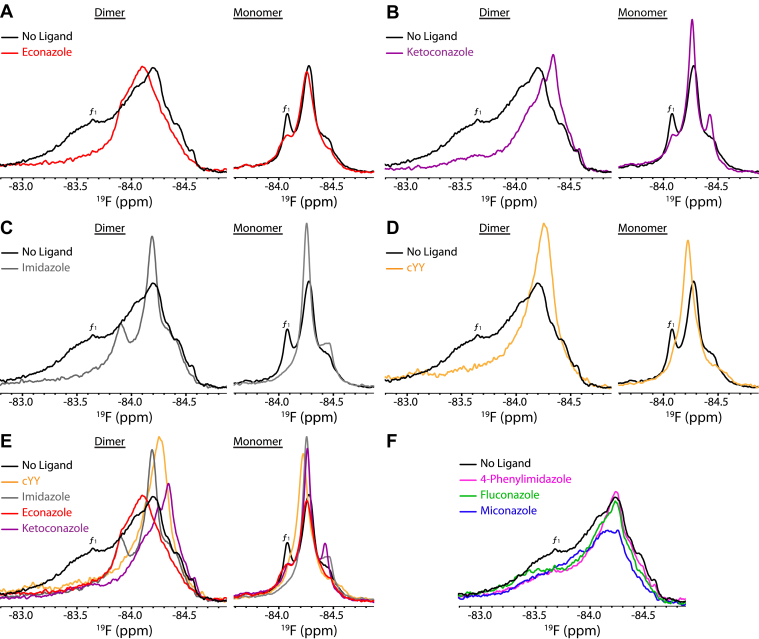
**Effect of various azoles on**^**19**^**Flabeled CYP121A1.** The econazole- (*A*/*red*), ketoconazole- (*B*/*purple*), imidazole- (*C*/*gray*), and cYY- (*D*/*yellow*) bound dimer and monomer. *E*, the overlay of preceding panels. *F*, ligand-free (*black*) and 4-phenylimidazole- (*pink*), fluconazole- (*green*), and miconazole- (*blue*) bound dimer. ^19^F, fluorine.

The inhibitor-bound spectra for CYP121A1 dimers are shown in Figure 7 (left panels). For comparison, they are also overlaid with the ligand-free spectrum (black trace) and are shown alongside overlays of the imidazole-bound spectrum (Fig. 7*C*) and the cYY-saturated spectrum (Fig. 7*D*). We observed that the ^19^F-NMR spectra display considerable perturbations from the ligand-free data. In all spectra, similar to the cYY-titration, the broad ligand-free peak *f*_*1*_ is not detected, suggesting that this resonance may represent open conformations of the FG loop. The central resonance peak *f*_*2*_ is most affected, with econazole inducing a downfield shift to −84.11 ppm (Fig. 7*A*) and ketoconazole inducing an opposite upfield shift to −84.34 ppm (Fig. 7*B*). Interestingly, other azoles (4-phenylimidazole, miconazole, and fluconazole) had a minimal effect on the shape or intensity of the NMR spectra (Fig. 7*F*).

Of note, we also observed that for each ligand that causes a unique ^19^F perturbation, the contours of the broad ligand-free spectra (see black trace in Fig. 7*E*) contain shoulders or minor peaks that align with the major peaks from each of the azole-bound spectra. The fact that these ligand-bound conformations exist to some extent without the ligand present suggests that the FG loop samples multiple ligand-bound conformations and that ligand binding may redistribute the equilibrium between these states. This notion is also supported by the observation that the ketoconazole-bound spectrum displays a minor peak at −84.25 ppm that closely aligns with the major peak from the cYY-bound spectrum, indicating that the ligand-bound conformations are not entirely ligand specific and may represent distinct energetically favorable arrangements of the FG loop.

Next, we compared the ligand-bound spectra with spectra of the I166A_I180A mutant (right panels in Fig. 7, *A–D*). Dialysis of the monomer into buffers containing various ligands produced Soret peaks of 421 nm and 420 nm for samples containing ketoconazole and econazole, respectively. The greater absorption changes relative to that of the dimer are consistent with higher affinity of the monomeric enzyme for nonspecific ligands (18). In stark contrast to the dimeric protein, each azole compound produced a similar perturbation in the monomeric protein, with the major peaks overlapping near −84.26 ppm. Only the cYY-bound conformation (orange trace in the Fig. 7*E*, right panel) results in a perturbation at a unique chemical shift value. This comparison highlights the direct structural role that the dimer interface plays in facilitating ligand-binding in CYP121A1.

## Discussion

Macromolecular solution NMR has previously been deployed to detect substrate and redox partner-induced changes in CYP conformation. However, its broader application has been limited to bacterial systems in which the CYP is amenable to monomerization (CYP101) (30, 31) or mammalian systems in which the enzyme remains a monomer after extraction from the membrane (CYP17A1) (6). Therefore, multimerization of CYPs, which is commonplace, remains a challenge for these studies because of increased transverse relaxation and spectral crowding of NMR spectra.

^19^F-NMR has increasingly been used to study macromolecular systems that, due to their size, are not tractable using uniform ^15^N labeling (32, 33, 34). Recent examples include, but are not limited to, studies of conformational assemblies of the A_2A_ G protein–coupled receptor (32) and dynamics in the Hsp90 chaperone protein (33). Here, we demonstrate the utility of ^19^F-NMR in the study of the Mtb essential enzyme CYP121A1, which forms a functional homodimer of approximately 90 kDa in solution (18). Currently, more than 50 crystallographic structures of CYP121A1 have been reported in a variety of ligand-bound and ligand-free states (13, 35, 36). A comparison of multiple ligand-bound structures, including a cYY-bound structure, indicates the existence of an overlapping binding mode in a region of the active site that is approximately 6 Å from the heme and directly adjacent to the F and G α-helices. The F and G α-helices and the intervening FG loop have a well-established role in ligand recognition and ligand binding. For example, in the thermophilic enzyme CYP119, aromatic residues on the FG loop reposition up to 12 Å, extending into the active site on addition of the ligand (22, 23). Further examples include the considerable displacement of the FG loop on binding the ligand in the mammalian drug-metabolizing enzymes CYP2B6 and CYP3A4 (19, 20, 21). In this context, the invariability of the FG loop position in ligand-bound CYP121A1 structures is particularly noteworthy.

We previously used the thiol-reactive label BTFA in combination with an S171C mutation to determine that the FG loop in CYP121A1 is structurally heterogeneous. Moreover, a comparison of spectra of the dimer and an engineered monomer (I166A_I180A) suggests that the adjacent protein dimer interface confers structural rigidity at the FG loop, with the engineered monomer displaying more disorder at this site (18). In this study, addition of incremental concentrations of cYY into multiple ^19^F-labeled samples of CYP121A1 and S171C results in the formation of a single major peak in both the monomer and the dimer (Fig. 3), suggesting that cYY reduces FG loop heterogeneity and is consistent with saturation of the binding site. A common feature of nearly all ligand-bound spectra in this study is the disappearance of the broad peak *f*_*1*_ in the dimer and the corresponding, albeit narrower, peak *f*_*1*_ in the monomer. This may indicate that this downfield resonance of the FG loop represents open conformations of the enzyme, which are then redistributed into upfield, closed assemblies upon addition of the ligand. We also observed that in the case of the dimer, the cYY-bound peak is already present as a substrate-free peak that forms a shoulder upfield of the broader *f*_*2*_ *peak*. This indicates that the conformational equilibrium of the FG loop already samples the substrate-bound conformation before substrate binding. This presampling was also observed in the dimer spectra of various CYP inhibitors (Fig. 7) but was only observed for the monomeric protein when in the presence of the dipeptides cYF and cYW (Fig. 6). In aggregate, these data directly implicate the dimer interface in facilitating ligand recognition and ligand binding.

The ability to monitor substrate binding from the perspective of the protein also allowed us to probe the specificity criteria for cYY recognition. Data collected in the presence of the dipeptide cYA failed to produce a change in the NMR spectrum (Fig. 6). Accordingly, cYA is a poor substrate that interacts only weakly *via* the spectral binding assay (29). A more interesting comparison is that of cYF and cYW, which preserve the two aromatic side chains but alter the positioning and composition of hydrogen bonding within the active site. Although cYF is not metabolized by CYP121A1 (highlighting the importance of the tyrosine hydroxyl group), cYW is actually a modest substrate with nearly half of the substrate depletion achieved with cYY (29). Despite this, the ^19^F-NMR spectrum in the presence of cYW overlays closely with that of the inactive cYF (Fig. 6). This indicates that the tyrosine that both compounds have in common is oriented toward the FG loop (consistent with their position in the cocrystal structure) and that loss of catalysis only occurs because of differences in hydrogen bonding at the aromatic side chain that is oriented away from the FG loop and toward the heme. Moreover, the comparison between cYW- and cYY-containing spectra demonstrates the importance of the positioning and orientation of the hydrogen-bonding group in this heme-facing side chain in cYY binding.

Interestingly, cYY binding by ^19^F-NMR occurs at a slow chemical exchange and appears to be largely saturated by a 1:2 ratio of the enzyme to ligand (Fig. S3). In contrast, absorbance measurements at lower concentrations and using the same ratios of the enzyme and ligand do not induce significant displacement of the heme-coordinated water molecule (Fig. 4*B*). This disagreement in binding data appears to be due, in part, to a concentration effect (Fig. 4*G*) in which the enzyme and substrate interaction is enhanced at higher protein concentrations despite the low relative amount of cYY present. Although interesting, this concentration effect makes it difficult to directly compare substrate binding as measured by each technique. There is nonetheless some evidence that binding by ^19^F-NMR does not entirely correlate with spectral binding data. For instance, our attempt to remove cYY by first diluting and then dialyzing the sample was not entirely successful (Fig. 4) because the ^19^F-NMR spectrum suggests that cYY remains largely bound to the protein. Additional evidence is provided by a comparison of the interaction of cYY with the dimer and with the I166A_I180A double mutant. Here, we observed that ^19^F-NMR spectra for either form of the enzyme are saturated at a similar point (Figs. 3 and S3), whereas spectral binding data from our previous study reveal a significant decrease in the affinity of the monomer for cYY. To be sure, there is additional evidence for multistep binding of ligands in CYP121A1. For example, the use of isothermal titration calorimetry indicated a 10- to 100-fold higher affinity for select substrate analogs than was measured by spectral binding, with binding of certain compounds only detectible by isothermal titration calorimetry (15). Other studies relied on stopped-flow absorbance measurements of cYY binding that fit best to a two-exponential model (37) and in combination with electron paramagnetic resonance, propose a low-spin, enzyme–substrate intermediate before dissociation of the heme-coordinated water ligand (38). Follow-on studies of cYY binding at lower protein concentrations will be required to determine whether a high-affinity binding site is present.

The ^19^F-NMR data presented in this work, combined with our previously reported model of the CYP121A1 functional homodimer, inform a model of CYP121A1 function in which substrate binding occurs *via* a specific recognition event near the homodimer interface. While the FG loop may not be directly involved in substrate binding, it appears to be in a position that is sensitive to the presence of cYY. In the modeled interface, the FG loop of each molecule is oriented on opposite sides of the complex. In theory, this arrangement allows for two separate binding events to occur simultaneously, although the question of cooperativity between molecules remains to be addressed. In summary, these findings illustrate the importance of the homodimer interface in maintaining substrate specificity and function in CYP121A1. This work also demonstrates the utility of ^19^F-NMR as an additional tool to investigate substrate binding and conformational changes in the monomeric and multimeric CYPs.

## Experimental procedures

### Protein production

Plasmid containing the CYP121A1 gene in a pET-11a vector was a gift from the laboratory of Dr Andrew Munro (University of Manchester). The modification of the plasmid to incorporate a poly-histidine tag and mutagenesis was procured from GenScript. The expression and purification of WT and mutant CYP121A1 were carried out as described previously (18), with the following modifications. Cultures were grown in LB at 220 rpm and 37 °C until an A_600_ of 0.8 was reached. After induction with IPTG (1 mM) and δ-aminolevulinic acid (0.5 mM), cultures were incubated at 26 °C for 48 h. The expression and purification of bovine adrenodoxin reductase and bovine adrenodoxin were carried out as previously described (7).

### Spectral binding

Ligand binding was measured by monitoring changes in the UV–Vis absorption spectra of CYP121A1 in a 1-cm quartz cuvette on a dual-beam Shimadzu 2700 spectrophotometer (https://ssi.shimadzu.com/). The spectra were monitored between 250 nm and 700 nm. Difference spectra were generated by subtracting ligand-bound spectra from ligand-free spectra. To determine the effect of the ^19^F label on ligand binding, stocks of cYY (a gift from the laboratory of Dr Claire Simons, Cardiff University) were dissolved in dimethyl sulfoxide (DMSO) and titrated into a 1 μM solution of CYP121A1 in 10 mM potassium phosphate, pH 7.4. The titration was carried out in triplicate at 25 °C with a 10-min incubation period between titration points to facilitate binding. The dissociation constants were calculated by plotting the ligand concentration against the difference spectra, which showed a high-spin (type-I) transition in response to cYY. Data fitting was carried out using Prism GraphPad v7.05 and using a single binding mode equation as described previously (7).

### ^19^F-NMR sample preparation

Labeling was carried out as described previously (18). The Ni-NTA elution fraction was diluted to 2 μM in 50 mM Tris HCl, pH 7.4, and 300 mM NaCl. This was supplemented with 10 mM BTFA and 5 mM DTT and incubated overnight at 4 °C without agitation. Optimization of the protein concentration during labeling was required because of precipitation when labeling was carried out at high concentrations. Unreacted BTFA and DTT were then removed by gel filtration, and the protein was exchanged into the NMR buffer (50 mM potassium phosphate, pH 7.4, 50 mM NaCl, and 10% D_2_O) using a 10-kDa molecular weight cutoff filter. Labeling specificity was determined using MS analysis of labeled S171C protein (Supplemental Methods) in which BTFA labeling was confirmed at 0.1% and 9% for Cys-51 and S171C, respectively, with no modifications detected at Cys-147 and Cys-345. Labeling of the I166A_S171C_I180A protein was confirmed at 28% at S171C, with no significant labeling detected elsewhere. Despite lower-than-expected labeling efficiency, spectra from multiple labeling batches showed reproducible signal intensities indicating that labeling efficiency is consistent.

For cYY titrations, the samples were supplemented with respective concentrations of cYY dissolved in DMSO, with the total volume of DMSO not exceeding 1.4% in all the samples. The azole-bound NMR spectra were acquired from samples in which the labeled protein had been diluted to approximately 4 μM and dialyzed overnight against 1 l of the NMR buffer supplemented with the respective azole compound present at the aqueous supersaturating concentrations of 200 μM. Imidazole-containing samples were exchanged into the NMR buffer containing incremental concentrations of the ligand and corrected for pH changes. To ensure the consistency of titration data, all samples used for ligand titrations were prepared from the same batch of ^19^F-labeled proteins. For NMR data collected on a 1:20 ratio of CYP121A1:cYY, a lower concentration of the protein (100 μM) was used. A separate set of samples designed to detect dissociation of cYY was prepared as follows. NMR samples that either contained 250 μM cYY or were substrate free were diluted to 20 ml in the NMR buffer and then dialyzed against 1 L of the substrate-free NMR buffer at 4 °C for 3 h. The samples were then concentrated to 250 μM and prepared for NMR as described above.

### ^19^F-NMR data acquisition and analysis

Spectra of CYP121A1 were acquired at 25 °C on an Agilent 400 MHz spectrometer with a 30° pulse angle, 1-s delay, and −84 ppm transmitter offset frequency for 10,000 scans per experiment. The temperature was increased in 5 °C increments for the temperature titration experiment, with 2500 scans collected for each sample. The data were processed and analyzed in Bruker, TopSpin, version 4.0.6, with 6-Hz line broadening applied. The binding constants were approximated from NMR titration data with cYY using an equation (Equation 1) adapted from the analysis of chemical-shift perturbations (39) to instead measure changes in peak intensity for an interaction occurring at a slow chemical exchange. Briefly, observed and maximum chemical-shift perturbations are substituted by observed (Δ*I*_obs_) and maximum (Δ*I*_max_) intensity measurements. Relative peak intensity data were quantified for each titration point at the saturated peak values of −84.22 ppm (dimer) and −85.97 ppm (monomer). The intensity data were plotted against cYY concentrations and the resulting plot fit to Equation 1 in Prism v7.05.

(1) $$\Delta I_{\text{obs}}=\;\Delta I_{\max}\left\{\left({\left[P\right]}_{t}+{\left[cYY\right]}_{t}+Kd\right)-{\left[{\left({\left[P\right]}_{t}+{\left[cYY\right]}_{t}+Kd\right)}^{2}-\;4{\left[P\right]}_{t}{\left[cYY\right]}_{t}\right]}^{1/2}\right\}/2{\left[P\right]}_{t}$$

## Data availability

All processed one-dimensional NMR spectra are available upon request. All remaining data are contained within the article and supporting information.

## Supporting information

This article contains supporting information (18, 40, 41, 42).

## Conflict of interest

The authors declare that there are no conflicts of interest with the contents of this article.
