# Supplementary material for: 19F-NMR reveals substrate specificity of CYP121A1 in Mycobacterium tuberculosis
Source: J Biol Chem. 2021 Oct 8;297(5):101287. doi: 10.1016/j.jbc.2021.101287 (PMC8571521; doi:10.1016/j.jbc.2021.101287)
Supplement: Supporting information [file mmc1.pdf]

SUPPORTING INFORMATION

^{19}F-NMR reveals substrate specificity of CYP121A1 in *Mycobacterium tuberculosis*

Christopher S. Campomizzi, George Ghanatios, D. Fernando Estrada

Department of Biochemistry, Jacobs School of Medicine and Biomedical Science, University at Buffalo, Buffalo, NY 14203, USA.

TABLE OF CONTENTS

Supplemental Figures S-2

Figure S1. Effect of BTFA labeling on CYP121A1 function and substrate interaction .. S-2

Figure S2. Concentration effect on ^{19}F spectra S-2

Figure S3. cYY binding measured by ^{19}F-NMR of the FG-loop S-3

Methods S-3

CYP121A1 functional assays S-3

Quantification of ^{19}F Labeling S-3

In-solution trypsin digestion........ S-3

Protein Identification by nano LC/MS/MS Analysis........ S-4

Data Analysis........ S-4

References S-5

SUPPLEMENTAL FIGURES

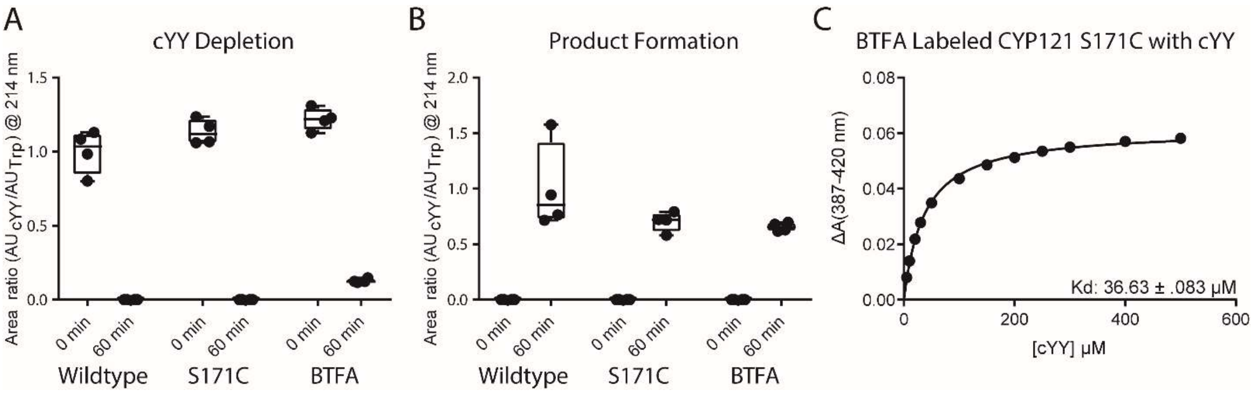

Figure S1. Effect of BTFA labeling on CYP121A1 function and substrate interaction. cYY depletion (A) and product formation (B) for wildtype, S171C, and BTFA labeled S171C. cYY binding via UV-visible spectroscopy for BTFA labeled S171C.

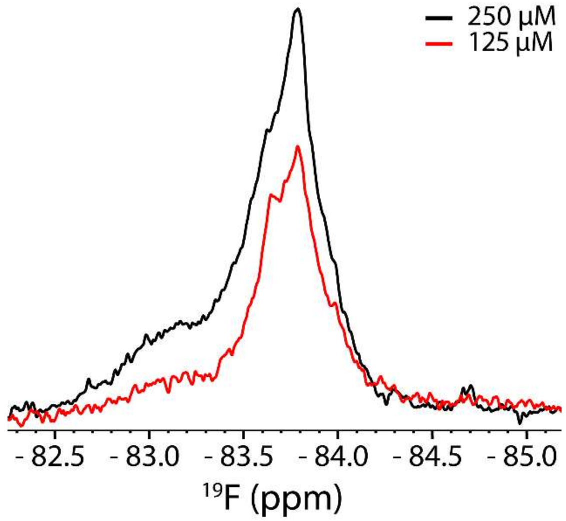

Figure S2. Concentration Effect on ^{19}F spectra. 250 μM (black trace) and 125 μM (red trace) of BTFA-labeled CYP121A1 S171C.

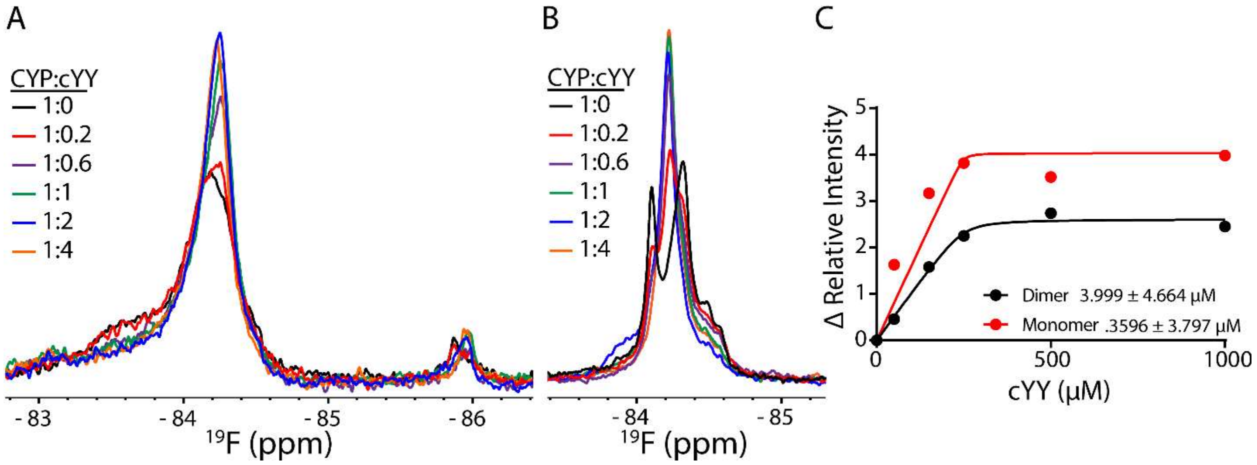

Figure S3. cYY binding measured by ^{19}F-NMR of the FG-loop. Complete overlay of ^{19}F cYY titration data for dimeric (A) and monomeric (B) CYP121. Quantification of cYY binding by ^{19}F-NMR (C) giving K_D values of 3.999 ± 4.664 µM for the dimer and 0.3596 ± 3.797 µM for the monomer.

METHODS

CYP121A1 functional assays – CYP121A1 functional assays were run using endpoint cYY depletion and with a reconstituted system that included Adx and AdR from bovine. The protocol is as previously described with minor modifications (18). The reaction mixture contained 5 µM CYP121A1 (quantified using initial CO-bound peak at 450 nm), 15 µM Adx, 5 µM AdR, and 150 µM substrate in 150 µL of 50 mM Tris HCl (pH 7.4). After preincubated at 30 °C for 5 minutes, the reaction was initiated with 1 mM NADPH. Following incubation at 30 °C for 60 minutes, the reactions were quenched by addition of 16.7 µL of 20% nitric acid, and 5 µM tryptophan was added as an internal reference. 50 µL of the quenched reaction was combined with 50 µL of acetonitrile, and centrifuged at 10,000 rpm for 30 minutes. 20 µL of the supernatant was diluted into 180 µL of 10% acetonitrile and 0.1% formic acid in water. Substrates and product peaks were resolved on a Poroshell 120 EC-C18 column (4.6 mm × 250 mm) (Agilent) on an Agilent 1260 Infinity II liquid chromatography system. All reactions were carried out in quadruplicate.

Quantification of ^{19}F Labeling

In-solution trypsin digestion – In solution digestion for each sample was performed on an S-Trap micro spin column (ProtiFi, Huntington, NY, USA) following an Strap protocol as described previously, with slight modification (40,41). About five micrograms of proteins in 25 µL buffer containing 50mM TEAB pH 8.5, 6M Urea, 2M Thiourea, 1% SDS were reduced with 15 mM Dithiothreitol (DTT) for 1 h at 34 °C, alkylated with 50 mM iodoacetamide for 1 h in dark and then quenched with a final concentration of 46 mM DTT. After quenching, 12% phosphoric acid was added to each sample for a final concentration of 1.2%, which was followed by 1:7 dilution (v/v) with a solvent containing 90% methanol and 0.1M TEAB pH 8.5. Each of the resulting samples was then placed into a spin column and centrifuged 3000g for 30 sec, then washed

three times with 150 µl 90% methanol, 0.1 M TEAB pH 8.5. Digestion was performed by adding 25 µl trypsin (20 ng/µl) at 1:10 w/w (trypsin:proteins) in 50 mM TEAB pH 8.5 to the top of the spin column. The spin columns were incubated overnight (16 hr) at 37 °C. Following incubation, the digested peptides were eluted off the S-trap column sequentially with 40 µl each of 50 mM TEAB pH 8.5 followed by 0.2% formic acid and finally, 50% acetonitrile, 0.2% formic acid. Three eluates with eluted peptides were pooled together and evaporated to dryness by a Speedvac SC110 (Thermo Savant, Milford, MA).

Protein Identification by nano LC/MS/MS Analysis – The tryptic digests were reconstituted in 57 µL of 0.5% formic acid (FA) for nanoLC-ESI-MS/MS analysis. The analysis was carried out using an Orbitrap FusionTM TribridTM (Thermo-Fisher Scientific, San Jose, CA) mass spectrometer equipped with a nanospray Flex Ion Source, and coupled with a Dionex UltiMate 3000 RSLCnano system (Thermo, Sunnyvale, CA) (40,42). The peptide samples (2 µL) were injected onto a PepMap C-18 RP nano trapping column (5 µm, 100 µm i.d x 20 mm) at 20 µL/min flow rate for rapid sample loading and then separated on a PepMap C-18 RP nano column (2 µm, 75 µm x 25 cm) at 35 °C. The tryptic peptides were eluted in a 60 min gradient of 5% to 35% ACN in 0.1% formic acid at 300 nL/min., followed by a 7 min ramping to 90% ACN-0.1% FA and an 8 min hold at 90% ACN-0.1% FA. The column was re-equilibrated with 0.1% FA for 25 min prior to the next run. The Orbitrap Fusion was operated in positive ion mode with spray voltage set at 1.9 kV and source temperature at 275°C. External calibration for FT, IT and quadrupole mass analyzers was performed. In data-dependent acquisition (DDA) analysis, the instrument was operated using FT mass analyzer in MS scan to select precursor ions followed by 3 second "Top Speed" data-dependent CID ion trap MS/MS scans at 1.6 m/z quadrupole isolation for precursor peptides with multiple charged ions above a threshold ion count of 10,000 and normalized collision energy of 30%. MS survey scans at a resolving power of 120,000 (fwhm at m/z 200), for the mass range of m/z 375-1575. Dynamic exclusion parameters were set at 50 s of exclusion duration with ±10 ppm exclusion mass width. All data were acquired under Xcalibur 4.4 operation software (Thermo-Fisher Scientific).

Data analysis – The DDA raw files with MS and MS/MS were subjected to database searches using Proteome Discoverer (PD) 2.4 software (Thermo Fisher Scientific, Bremen, Germany) with the Sequest HT algorithm. The PD 2.4 processing workflow containing an additional node of Minora Feature Detector for precursor ion-based quantification was used for protein identification and relative quantitation of identified peptides and their modified forms. The database search was conducted against an *E.coli* database with added two sequences for targeted proteins: CYP121 S171C and I166A_S171C_I180A . The peptide precursor tolerance was set to 10 ppm and fragment ion tolerance was set to 0.6 Da. Variable modification of methionine oxidation, deamidation of asparagines/glutamine, 3-bromo-1,1,1-trifluoacetone of cysteine, acetylation on protein N-terminus and fixed modification of cysteine carbamidomethylation, were set for the database search. Only high confidence peptides defined by Sequest HT with a 1% FDR by Percolator were considered for confident peptide identification.

All identified peptides containing a modified Cys with 3-bromo-1,1,1-trifluoacetone by PD 2.4 were further verified by manual inspection of the relevant MS and MS/MS spectra. Relative quantitation of identified 3-bromo-1,1,1-trifluoacetone modified peptides and their native counterparts within single samples was determined by manual extraction ion chromatograms

(XICs). The XICs of each peptide (and its modified form) with different charge states for each sample were obtained based on MS precursor m/z with mass tolerance of 5 ppm in Xcalibur software. The relative quantitation (occupancy rate) of the modified form with 3-bromo-1,1,1-trifluoacetone in a tryptic peptide was calculated with the peak area of XICs, based on the assumption that the ionization efficiency for the modified peptides and their corresponding native forms is the same.

REFERENCES

18. Kumar, A., Campomizzi, C. S., Jay, N., Ferguson, S., Scheffler, E. J., Lioi, J., Tu, C., Qu, J., Simons, C., and Estrada, D. F. (2021) Surface hydrophobics mediate functional dimerization of CYP121A1 of Mycobacterium tuberculosis. *Sci. Rep.* **11**, 394
40. Yang, Y., Anderson, E., and Zhang, S. (2018) Evaluation of six sample preparation procedures for qualitative and quantitative proteomics analysis of milk fat globule membrane. *Electrophoresis* **39**, 2332-2339
41. Zougman, A., Selby, P. J., and Banks, R. E. (2014) Suspension trapping (STrap) sample preparation method for bottom-up proteomics analysis. *Proteomics* **14**, 1006-1000
42. Harman, R. M., He, M. K., Zhang, S., and GR, V. D. W. (2018) Plasminogen activator inhibitor-1 and tenascin-C secreted by equine mesenchymal stromal cells stimulate dermal fibroblast migration in vitro and contribute to wound healing in vivo. *Cytotherapy* **20**, 1061-1076
